# Discovery of Innate Immune Response mRNAs That Are Impacted by Structure-Specific Oral Baker’s Yeast Beta Glucan Consumption

**DOI:** 10.3390/biotech14010004

**Published:** 2025-01-13

**Authors:** Brian K. McFarlin, John H. Curtis, Jakob L. Vingren, David W. Hill, Elizabeth A. Bridgeman

**Affiliations:** 1 Applied Physiology Laboratory, University of North Texas, Denton, TX 76203, USA; john.curtis@unt.edu (J.H.C.); jakob.vingren@unt.edu (J.L.V.); david.hill@unt.edu (D.W.H.);; 2 Department of Biological Sciences, University of North Texas, Denton, TX 76203, USA

**Keywords:** trained innate immunity, innate immune priming, NanoString, inflammation, infection

## Abstract

The study of nutritional compounds with the potential to train the innate immune response has implications for human health. The objective of the current study was to discover by what means 6 weeks of oral baker’s yeast beta glucan (BYBG) supplementation altered the mRNA expression of genes that reflect innate immune training in the absence of a physical stressor. Nineteen adults were randomly assigned to either a Wellmune^®^ BYBG or Placebo for 6 weeks. BYBG uniquely altered the expression of 40 mRNAs associated with Dectin-1 and trained innate immunity, the innate immune response, the pathogen-associated (PAMP) and damage-associated molecular pattern (DAMP), and the inflammatory response. The observed changes were classified as immune training rather than immune priming due to the progressive increase in the expression of myeloid immune-associated mRNA. Combined with the findings of previous research, the findings of the present study support the claim that oral BYBG supplementation may be associated with trained innate immunity during resting homeostasis. Further, the key findings associated with BYBG may reflect improved responsiveness to future infection (exogenous) and/or sterile-inflammatory (endogenous) challenge.

## 1. Introduction

The innate immune system does not have memory capacity but can be trained to respond to non-self antigens [1,2,3,4,5,6,7]. Trained innate immunity was initially reported in patients who received the Bacille Calmette–Guerin (BCG) vaccine [1,3,5]. Fortunately, further study of trained innate immunity has revealed that other naturally occurring compounds, such as baker’s yeast beta glucan (BYBG), can be used to train the innate immune system [8] in a minimally invasive manner compared to vaccines that require doctor/pharmacy visits and needles. The majority of the published literature has identified that ligation of Dectin-1 via orally consumed BYBG is the primary means by which innate immune training occurs [9,10,11,12,13]; however, it is plausible that other, yet to be identified pathways may also be involved. Oral BYBG is absorbed via the Peyer’s patch in the gastrointestinal tract, where it interacts with innate immune cells, resulting in innate immune training.

Prior to the knowledge that certain BYBGs act via an innate immune training mechanism, BYBG was traditionally studied by exposing participants to an acute or chronic physical stressor (e.g., exercise, physically demanding work) [14,15,16,17,18,19]. Physical stress increases susceptibility to opportunistic infection due to transient disruption of the normal immune response and surveillance [16,17,19,20]. In these previous studies, our laboratory and others demonstrated that oral BYBG supplementation improved various aspects of the innate immune response and reduced infection risk [14,15,16,17,18]. While previous studies have yielded valuable information regarding the application of BYBG supplementation to human health, additional research is needed to determine new biomarkers that may be useful for detecting the effects of BYBG both at rest and in response to a physical stressor.

One advantage of previous BYBG physical stressor models is that they are associated with moderate effect sizes, increasing the likelihood of detecting BYBG response biomarkers. However, previous studies that attempted to study the effects of BYBG under resting conditions tended to have small effect sizes for biomarkers, making it difficult to detect a change. Also, once the innate immune system has adapted to BYBG, it tends to return to a homeostatic setpoint (i.e., baseline) that is challenging to measure using traditional outcome measures. It is reasonable to speculate that the use of more sensitive molecular biomarkers may be required to detect a BYBG response at rest. Molecular techniques have long been used by the pharmaceutical industry for drug discovery because mRNA biomarkers tend to be more sensitive to perturbation than traditional protein biomarkers are [21,22,23,24]. Whereas mRNA and protein biomarkers may reveal different systemic responses, both are useful for understanding the effect of a treatment. In our laboratory, we demonstrated that multiplex mRNA detection allows robust low-end detection with low measurement error in human samples [25,26,27,28,29,30]. This approach is supportive of “discovery” outcomes such as in the present study.

In addition to detecting changes in individual mRNAs with precision, an advantage of mRNA biomarkers is the ability to complete annotated pathway enrichment. This analytical technique allows the integration of global mRNA changes to better understand the impact of an intervention on different immune response pathways. We speculated that using multiple mRNA biomarkers as outcome measures would allow us to identify a BYBG-trained innate immune signature in resting samples. While BYBG has been previously reported to cause innate immune training, the present study did not measure trained innate immunity per se. Rather, the present study sought to discover which innate immune associated mRNAs may change with BYBG. The purpose of the present study was to identify/discover a BYBG signature from healthy individuals over a period of 6 weeks of BYBG supplementation. We speculated that the BYBG signature would be defined by an integration of the changes in individual mRNAs and the enrichment of specific innate immune response pathways.

## 2. Materials and Methods

### 2.1. Ethics Approval and Consent to Participate

After the risks and benefits of participation were explained, all participants provided oral and written consent to participate in the present study. The study followed the latest Declaration of Helsinki, and all protocols were approved by the University of North Texas Institutional Review Board (IRB; Protocol code IRB-21-287; 1st approved on 10 August 2021). Participants were made aware that the results of the study would be published, but no individual data are presented in the present manuscript.

### 2.2. Experimental Model and Participants

A sample size analysis was conducted prior to the present study using previously published research by our laboratory [26,27,28,29,30]. In order to evaluate trained innate immunity at rest over a period of 6 weeks, an N = 5 in each condition (total N = 10) would result in 80% statistical power. To further increase statistical power, we enrolled N = 10 per condition. (total N = 20) and had N = 19 complete the entire study protocol, resulting in >90% statistical power. N = 10 dropped out of the study due to an inability to schedule their final study appointments.

Healthy adults (age 31 ± 4 y; BMI 25.4 ± 1.2 kg/m^2^) with no diagnosed metabolic or inflammatory disease were enrolled. Subjects were assigned to either Wellmune^®^ BYBG (Kerry, Inc., Beloit, WI, USA) or Placebo (maltodextrin) at random. The structure of a given BYBG strongly impacts its function; thus, we used a BYBG in this study that has well-documented structure-function claims [15,16,17,18,19,20,28]. BYBG daily intake increased progressively from 50 mg/day (weeks 1 and 2) to 125 mg/day (weeks 3 and 4) and 250 mg/day (weeks 5 and 6). This dose range and strategy was selected based on previously published literature as having similar effects [15,16,17,18,19,20,28]. While it may seem logical to use the same dose throughout, given similar previously reported outcomes with 50, 125, and 250 mg/day, it is likely the observed changes are related to duration of dosing rather than specific dose. The expression of each of the 730 different mRNAs was determined in the two groups at baseline and after 2, 4, and 6 weeks of supplementation. The data for the two groups were compared to determine the effect of BYBG supplementation. Daily supplements were provided in pill form to the participants in prepackaged blister packs. The emptied blister packs were returned to the study staff every 2 weeks, when new packs were provided, and blood was collected. Based on the number of returned empty blister packs, compliance with the supplementation protocol was 92%.

### 2.3. Blood Sample Collection

Before any supplementation (baseline) and after 2, 4, and 6 weeks of supplementation, participants reported to the laboratory between 0600 h and 0900 h following an overnight fast, except for water (>8 h), and abstention from exercise (>24 h). A PAXgene RNA stabilizing evacuated tube (PreAnalytiX, Hombrechtikon, Switzerland) was used to collect venous blood for total RNA isolation.

### 2.4. Total RNA Extraction and mRNA Expression Analysis

The analysis in this manuscript was completed as described in detail previously [27,29]. Briefly, PAXgene tubes were used to isolate RNA with an automated, spin column isolation method (QIAcube; Qiagen, Hilden, Germany). Isolated total RNA was analyzed for the expression of 770 mRNAs associated with human myeloid immunity (nCounter; NanoString, Seattle, WA, USA). Control adjustments and expression relative to internal controls was completed as described previously [27,29]. The data were analyzed with a HyperScale ROSALIND^®^ (https://rosalind.onramp.bio/, accessed on 1 June 2024) (ROSALIND, Inc., San Diego, CA, USA). The read distribution percentages, identity heatmaps, and sample multidimensional plots were generated as part of the quality control analysis. The NanoString criteria were used for normalization, fold changes, and *p* value calculations. ROSALIND^®^ follows the nCounter^®^ Advanced Analysis protocol of dividing counts within a lane by the geometric mean of the normalization probes from the same lane. 

### 2.5. False Positive/False Negative Control

False Discovery Rate (FDR) is a control for a Type I error that can occur when multiple comparisons are completed; however, the nature of multiplex mRNA expression analysis does not warrant universal corrections due to false negative inflation risk. Our FDR correction approach removed false positives that had an expression fold change between −0.5 and 0.5, had no BYBG treatment effect, and/or had no previously published studies linking the observed expression to the study key words. Of the initial 77 mRNA expressions that reached statistical significance, 37 mRNA expressions were removed using our false positive control procedures.

### 2.6. Data Reporting

Differential mRNA expression was calculated at all time points (i.e., baseline, week 2, week 4, and week 6) using placebo as a comparison point (i.e., setting placebo as a zero change). Specifically, mRNA was expressed as the log2-fold change from the corresponding placebo value to normalize the response to a 0 center and indicate the direction of expression (i.e., up- or down-regulated). Significance was set at *p* < 0.05 for all comparisons. After determining the significance of the differences, we performed a systematic review of the literature and used NanoString pathway annotations to assign each significant mRNA to one of three immune response pathways: (1) Dectin-1 and trained innate immunity, (2) innate immune response, and (3) damage-associated molecular pattern (DAMP), pathogen-associated molecular pattern (PAMP), and inflammatory response. Global pathway enrichment values were calculated to provide a global significance score, which is the overall differential expression of gene sets independent of the direction of change for each gene. Global significance scores > 1.0 for at least one time point were reported. As part of the analysis, routine quality control testing was conducted to ensure that the assay results were consistent with the manufacturer’s recommended ranges. The outcomes are presented as volcano plots, standard bar graphs, and bubble plots. The goal of these analyses was to identify both individual mRNAs and enriched pathways that collectively may reflect a BYBG signature.

## 3. Results

### 3.1. Overview of Results and Discussion

The key objective of the present study was to identify an mRNA expression signature in individuals supplemented with BYBG in the absence of a perturbation (i.e., exposure to infection, physical stressor, etc.). Once appropriate control for multiple comparisons and removal of false positives was completed, remaining, significant mRNAs were classified relative to the immune response pathway they were related to based on a literature search. This resulted in mRNAs being assigned to the following pathways: (1) Dectin-1 and trained innate immunity, (2) innate immune response, or (3) PAMP, DAMP, and the inflammatory response. The results and discussion were organized according to these response pathways to provide consistency for other researchers.

### 3.2. Names of mRNAs Significant with BYBG

The names of the mRNAs that reached significance with BYBG supplementation were as follows: S100 calcium-binding protein (S100A8), Thioredoxin (TXN), Bruton’s tyrosine kinase (BTK), -c chemokine receptor type 2 (CCR2), colony stimulating factor 3 receptor (CSF3R), interferon regulatory factor 5 (IRF5), interferon regulatory factor 7 (IRF7), mannose receptor c type 2 (MRC2), tumor necrosis factor superfamily member 8 (TNFRSF8; also known as CD30L), carcinoembryonic antigen-related cell adhesion molecule 1 (CEACAM1), Sequestosome-1 (SQSTM1), neutrophil cytosol factor 2 (NCF2), ribonuclease A family member 2 (RNASE2), GATA Binding Protein 1 (GATA1), MX dynamin-like GTPase 2 (MX2) and interferon-induced proteins with tetratricopeptide repeats (IFIT1), Signal regulatory protein A (SIRPA), Selectin P ligand (SELPLG), cadherin-1 (CDH1), C-X-C chemokine receptor (CXCR1), Natural killer cell group 7 sequence (NKG7), CD32, Myeloid differentiation primary response 88 (MYD88), Interferon regulatory factor 7 (IRF7), junctional adhesion molecule A (F11R), tissue transglutaminase (TG2), V-domain Ig suppressor of T-cell activation (VISTA), GTP-binding protein (RHOG), PYD and CARD domain containing (PYCARD), CD87, CD97, ADAM metallopeptidase domain 8 (ADAM8), and Indoleamine 2,3-dioxygenase 1 (IDO1).

**Figure 1:**
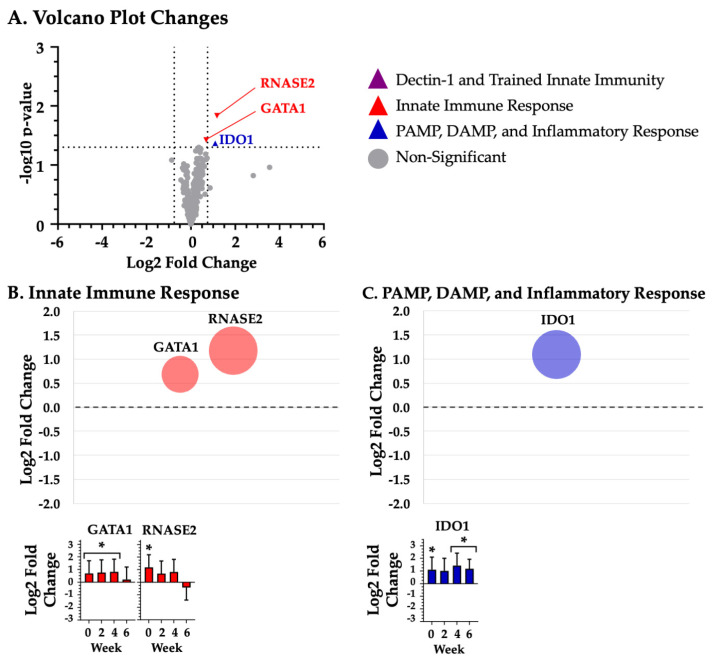
**No BYBG supplementation.** Differentially expressed BYBG mRNAs compared to placebo mRNAs prior to supplementation (baseline). Panel (**A**) represents a volcano plot of the differential gene expression between BYBG and placebo. Significantly changed mRNAs at baseline are represented by purple (dectin-1 and trained immunity), red (innate immune response), and green (PAMP, DAMP, and inflammatory response) triangles. Panel (**B**) and Panel (**C**) show a bubble plot and standard bar graphs of individual mRNAs associated with innate immune response, and PAMP, DAMP, and inflammatory response, respectively. * indicates significant mRNA expression change (*p* < 0.05).

**Figure 2:**
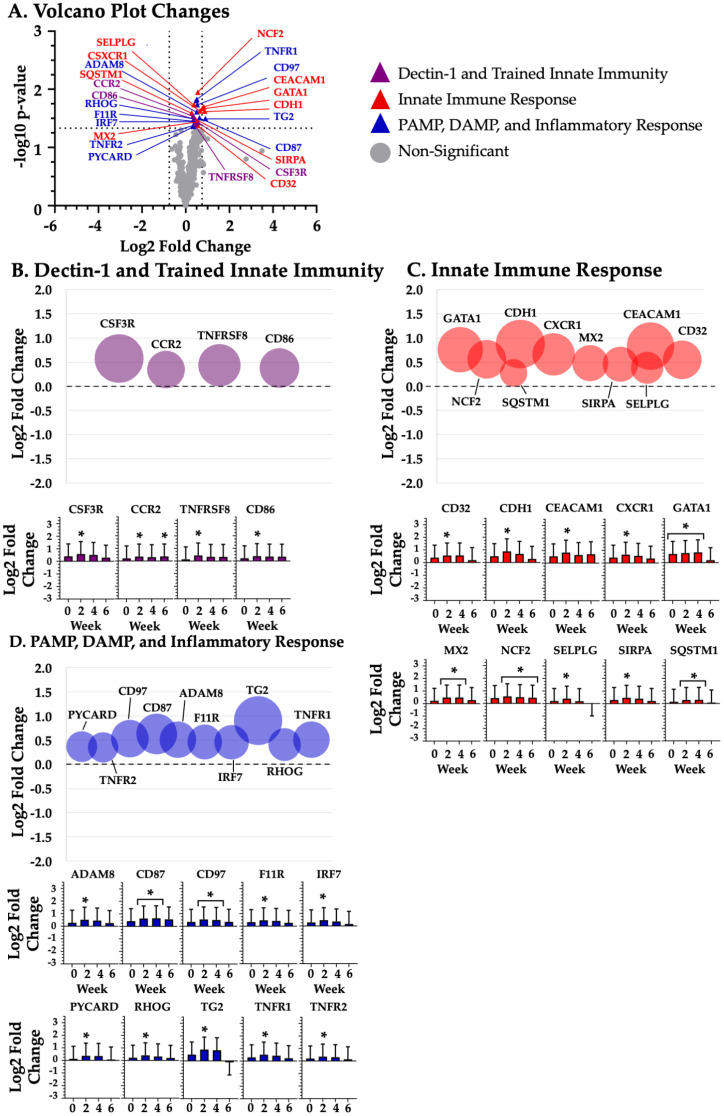
**Two weeks of BYBG supplementation.** Differentially expressed BYBG mRNAs compared to placebo mRNAs at 2 weeks. Panel (**A**) represents a volcano plot of the differential gene expression between BYBG and placebo. Significantly changed mRNAs at baseline are represented by purple (dectin-1 and trained immunity), red (innate immune response), and green (PAMP, DAMP, and inflammatory response) triangles. Panel (**B**), Panel (**C**), and Panel (**D**) show a bubble plot and standard bar graphs of individual mRNAs associated with dectin-1 and trained innate immunity, innate immune response, and PAMP, DAMP, and inflammatory response, respectively. * indicates significant mRNA expression change (*p* < 0.05).

**Figure 3:**
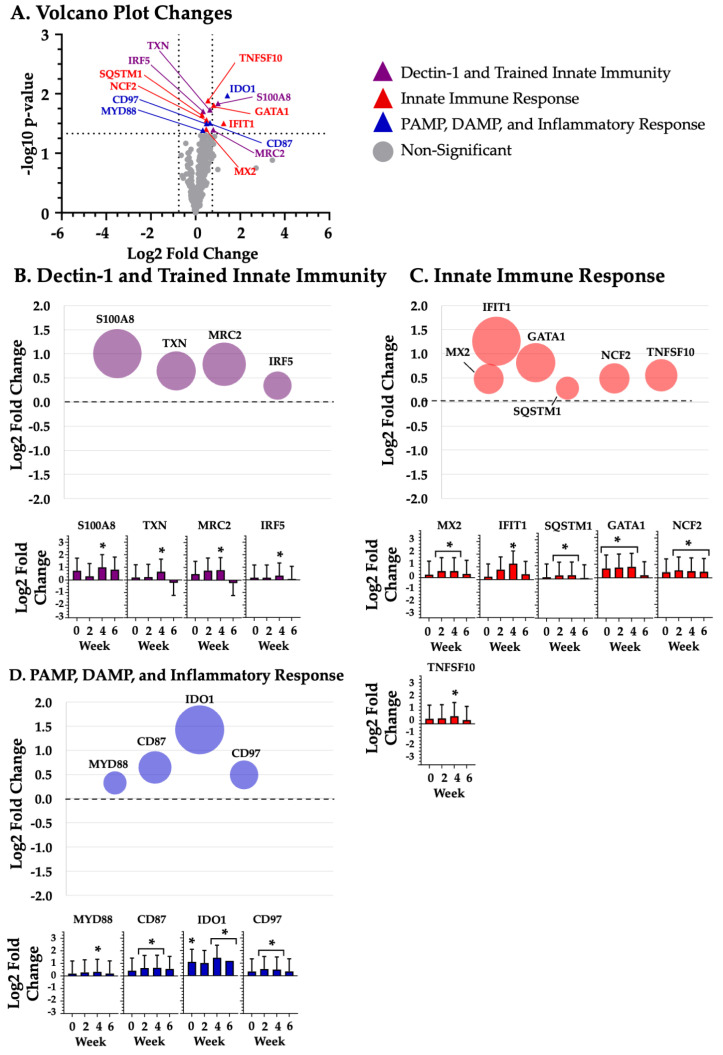
**Four weeks of BYBG supplementation.** Differentially expressed BYBG mRNAs compared to placebo mRNAs at 4 weeks. Panel (**A**) represents a volcano plot of the differential gene expression between BYBG and placebo. Significantly changed mRNAs at baseline are represented by purple (dectin-1 and trained immunity), red (innate immune response), and green (PAMP, DAMP, and inflammatory response) triangles. Panel (**B**), Panel (**C**), and Panel (**D**) show a bubble plot and standard bar graphs of individual mRNAs associated with dectin-1 and trained innate immunity, innate immune response, and PAMP, DAMP, and inflammatory response, respectively. * indicates significant mRNA expression change (*p* < 0.05).

**Figure 4:**
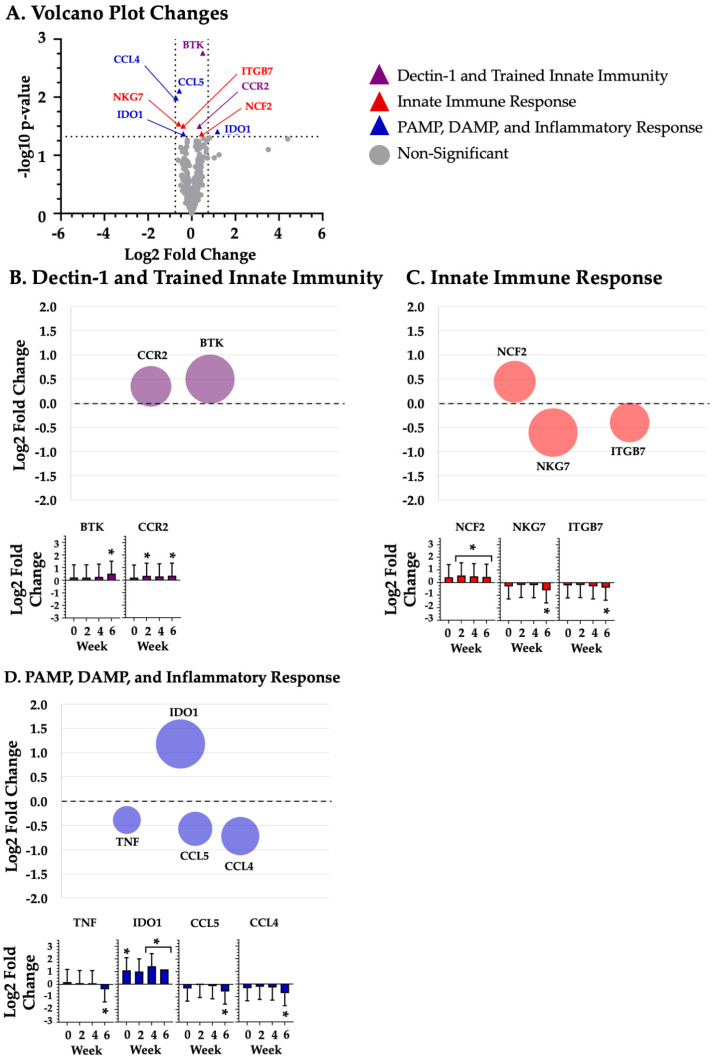
**Six weeks of BYBG supplementation.** Differentially expressed BYBG mRNAs compared to placebo mRNAs at 6 weeks. Panel (**A**) represents a volcano plot of the differential gene expression between BYBG and placebo. Significantly changed mRNAs at baseline are represented by purple (dectin-1 and trained immunity), red (innate immune response), and green (PAMP, DAMP, and inflammatory response) triangles. Panel (**B**), Panel (**C**), and Panel (**D**) show a bubble plot and standard bar graphs of individual mRNAs associated with dectin-1 and trained innate immunity, innate immune response, and PAMP, DAMP, and inflammatory response, respectively. * indicates significant mRNA expression change (*p* < 0.05).

Given the large number of changes at differing time points, mRNA grouped according the four study time points: baseline (Figure 1), week 2 (Figure 2), week 4 (Figure 3), and week 6 (Figure 4). 

### 3.3. Dectin-1 and Trained Innate Immunity

BYBG supplementation significantly (*p* < 0.05) increased the expression of the following nine Dectin-1 and trained innate immunity mRNAs (listed in alphabetical order): BTK (week 6; *p* = 0.002; Figure 4A,B), CCR2 (week 2; *p* = 0.032; week 6; *p* = 0.032; Figure 2A,B and Figure 4A,B), CD86 (week 2; *p* = 0.036; Figure 2A,B), CSF3R (week 2; *p* = 0.042; Figure 2A,C), IRF5 (week 4; *p* = 0.023; Figure 3A,B), MRC2 (week 4; *p* = 0.048; Figure 3A,B), S100A8 (week 4; *p* = 0.017; Figure 3A,B), TNFRSF8 (week 2; *p* = 0.044; Figure 2A,B), and TXN (week 4; *p* = 0.022; Figure 3A,B).

### 3.4. Innate Immune Response

BYBG supplementation significantly (*p* < 0.05) increased expression of the following 13 innate immune response mRNAs, listed in alphabetical order: CD32 (week 2; *p* = 0.047; Figure 2A,C), CDH1 (week 2; *p* = 0.027; Figure 2A,C), CEACAM1 (week 2; *p* = 0.022; Figure 2A,B), CXCR1 (week 2; *p* = 0.026; Figure 2A,C), GATA1 (baseline; *p* = 0.042; week 2; *p* = 0.024; week 4; *p* = 0.018; Figure 1A,C, Figure 2A,C and Figure 3A,C), IFIT1 (week 4; *p* = 0.037; Figure 3A,C), MX2 (week 2; *p* = 0.040; week 4; *p* = 0.046; Figure 2A,C and Figure 3A,C), NCF2 (week 2; *p* = 0.013; week 4; *p* = 0.032; week 6; *p* = 0.043; Figure 2A,C, Figure 3A,C and Figure 4A,C), RNASE2 (baseline; *p* = 0.016; Figure 1A,C), SELPLG (week 2; *p* = 0.021; Figure 2A,C), SIRPA (week 2; *p* = 0.037; Figure 2A,C), SQSTM1 (week 2; *p* = 0.029; week 4; *p* = 0.027; Figure 2A,C and Figure 3A,C), and TNFSF10 (week 4; *p* = 0.015; Figure 3A,C). BYBG also resulted in significantly decreased expression of two innate immune response mRNAs, listed in alphabetical order: ITGB7 (week 6; *p* = 0.031; Figure 4A,C) and NKG7 (week 6; *p* = 0.029; Figure 4A,C).

### 3.5. PAMP, DAMP, and the Inflammatory Response

BYBG supplementation significantly (*p* < 0.05) increased expression of the following 16 DAMP, PAMP, and inflammatory response mRNAs, listed in alphabetical order: ADAM8 (week 2; *p* = 0.027; Figure 2A,D), CCL4 (week 6; *p* = 0.010; Figure 4A,D), CCL5 (week 6; *p* = 0.008; Figure 4A,D), CD87 (week 2; *p* = 0.035; week 4; *p* = 0.036; Figure 2A,D and Figure 3A,D), CD97 (week 2; *p* = 0.021; week 4; *p* = 0.037; Figure 2A,D and Figure 3A,D), F11R (week 2; *p* = 0.040; Figure 2A,D), IDO1 (baseline; *p* = 0.048; week 4; *p* = 0.013; week 6; *p* = 0.039; Figure 1A,D, Figure 3A,D and Figure 4A,D), IRF7 (week 2; *p* = 0.039; Figure 2A,D), MYD88 (week 4; *p* = 0.048; Figure 3A,B), PYCARD (week 2; *p* = 0.049; Figure 2A,D), RHOG (week 2; *p* = 0.036; Figure 2A,D), TG2 (week 2; *p* = 0.037; Figure 2A,D), TNF (week 6; *p* = 0.043; Figure 2A,C), TNFR1 (week 2; *p* = 0.017; Figure 2A,D), TNFR2 (week 2; *p* = 0.047; Figure 2A,D), and VISTA (week 2; *p* = 0.018; Figure 2A,C).

### 3.6. Global Pathway Enrichment

Of the observed individual BYBG-induced changes in mRNA expression, the vast majority (38 of 40 significant mRNAs) occurred because of supplementation (i.e., they were not observed at baseline prior to supplementation). In addition to determining the changes in individual mRNAs, an enrichment analysis of immune pathways was also conducted, and the results are presented as a global significance score. This analysis provides a summary of the differential pathway mRNA expression, regardless of whether the mRNA was up- or down-regulated. These data show that the expression of genes in an entire pathway comprising many genes was changing. We identified eight pathways that were significantly impacted following BYBG supplementation (Table 1).

## 4. Discussion

### 4.1. Overview of Findings

In the present study, we sought to identify mRNAs whose expression may reflect trained innate immunity during a 6-week period of BYBG supplementation. This study was designed to complement previous research from our laboratory and others concerning the impact of oral BYBG supplementation on immune response capacity. The ultimate objective was to identify individual mRNAs and pathways that collectively may represent a BYBG signature. Previously published studies have used a physical stressor to elicit BYBG-induced responses but may have failed to fully elucidate all known effects [14,15,16,17,18]. The present study represents a novel approach in that we used a sensitive and precise multiplex mRNA expression methodology to track BYBG-induced changes in innate immune training absent a physical stressor. By examining the literature and the functions of the BYBG-altered mRNAs, we were able to associate (i) nine mRNAs with dectin-1 and trained innate immunity (BTK, CCR2, CD86, CSF3R, IRF5, MRC2, S100A8, TNFRSF8, and TXN); (ii) 15 mRNAs with innate immune response (CD32, CDH1, CEACAM1, CXCR1, GATA1, IFIT1, ITGB7, MX2, NCF2, NKG7, RNASE2, SELPLG, SIRPA, SQSTM1, and TNFSF10); and (iii) 16 mRNAs involved in the innate immune response (ADAM8, CCL4, CCL5, CD87, CD97, F11R, IDO1, IRF7, MYD88, PYCARD, RHOG, TG2, TNF, TNFR1, TNFR2, and VISTA). In total, the expression of 40 mRNAs was affected by BYBG supplementation over the 6-week period.

In addition to examining changes in individual mRNAs, our multiplex approach afforded us the opportunity to determine how BYBG supplementation impacted the enrichment of various innate immune response pathways. Specifically, we found that BYBG enriched eight immune response pathways. The most impacted pathways were interferon signaling, cell migration and adhesion, Toll-like receptor (TLR) signaling, antigen presentation, and complement activation (Table 1). Collectively, the enriched pathways and changes in the 40 individual mRNAs may reflect a signature of trained innate immunity associated with the structure of the BYBG used. There was certainly a time-dependent response to BYBG supplementation that changed over time, with the largest number of changes occurring at week 2. Based on the literature and known functional roles, we grouped the 40 individual mRNAs into three common innate immune functions.

### 4.2. Dectin-1 and Trained Innate Immunity

Dectin-1 is a receptor that recognizes BYBG [9,10] and may be associated with the development of trained innate immunity [31]. In the present study, we identified nine mRNAs that are associated with Dectin-1 activity [32,33,34,35,36]. CD86 functions along with CD80 (which was not measured in the present study) to contribute to antigen presentation [32,33]. An increase in the expression of CD86 in combination with BYBG may represent an immune priming effect for enhanced antigen presentation during the adaptive immune response in B cells. Our results are consistent with those of others, who reported that beta glucan increased CD86 expression via the Dectin-1 pathway [32,33].

The expression of S100 calcium-binding protein (S100A8), which is regulated by Dectin-1 pathway cytokines, contributes to gastrointestinal tract antimicrobial defense [34]. Thus, the increase in S100A8 expression partially confirms that the BYBG used in the present study may have activated Dectin-1. It is important to note that we did not observe a significant increase in S100A8 until week 4; however, since the current BYBG is known to interact rapidly with Dectin-1, it is unknown why the effect on S100A8 was delayed until week 4. Thioredoxin (TXN) is a damage-associated molecular pattern (DAMP) that signals through Dectin-1 to release IL-1β and through Dectin-2 to release IL-23 [35] and may be involved in sterile inflammation [36]. We observed a BYBG-associated increase in TXN expression at week 4, and given its known role as a DAMP, it is reasonable to speculate that this beneficial adaptation may allow for an improved immune response following sterile inflammation.

Bruton’s tyrosine kinase (BTK) plays a key collaborative role with Dectin-1 and VAV1 (which was not measured in the present study) to facilitate macrophage phagocytosis of the fungal pathogen *Candida albicans* [37]. In the present study, BYBG progressively increased BTK expression, reaching a peak and significance at week 6. Given its ability to directly impact Dectin-1 activity, it seems reasonable to speculate that we observed beneficial adaptation after 6 weeks of BYBG supplementation.

We also observed BYBG-associated changes in c-c chemokine receptor type 2 (CCR2), colony stimulating factor 3 receptor (CSF3R), interferon regulatory factor 5 (IRF5), interferon regulatory factor 7 (IRF7), mannose receptor c type 2 (MRC2), and tumor necrosis factor superfamily member 8 (TNFRSF8; also known as CD30L), all of which impact various aspects of trained innate immunity [38,39,40,41] but are not necessarily mediated by Dectin-1. Ligation of CSF3R mediates granulocyte maturation from naïve to trained cells [42]. The increased expression of CSF3R at week 2 with BYBG might reflect the accumulation of trained innate immunity, although the effect did not persist past 2 weeks.

CCR2 and TNFRSF8 collectively mediate the homing of proinflammatory monocytes to the pancreas and sites of peripheral sterile injury [39,40]. The observed increase in CCR2 (weeks 2 and 6) and TNFRSF8 (week 2) expression with BYBG may be indicative of the development of monocyte innate immune training. IRF5, IRF7, and MRC2 all exert similar effects on various aspects of macrophage phenotype switching and transcriptional regulation of innate immunity [38,41,43]. We found increased expression of IRF7 at week 2 and of both IRF5 and MRC2 at week 4. Given its role in innate immune response, it is logical that the expression of IRF7 was increased prior to the expression of IRF5 and MRC2, which exert more specific actions related to monocyte phenotype switching and cell-surface expression of CCR2.

Finally, we note that reduced MRC2 and increased Dectin-1 expression on myeloid cells are novel symptoms in patients with IBD [44]. Our finding that the mRNA expression of MRC2 was increased in the BYBG group may support the speculation that oral BYBG supplementation can not only cause trained innate immunity but also reduce the risk of other chronic inflammatory conditions via an optimized innate immune response.

### 4.3. Innate Immune Response

In the present study, the expression of 15 mRNAs linked to various aspects of innate immunity [45,46,47,48,49] was affected at various time points during 6 weeks of BYBG supplementation. Beta glucan has been reported to increase carcinoembryonic antigen-related cell adhesion molecule 1 (CEACAM1) expression in vitro [45], but to our knowledge, we are the first to report a similar effect in vivo. CEACAM1 expression plays a key role in the immune response to *Candida albicans* [50]. Thus, it is reasonable to speculate that the observed increase is a beneficial adaptation.

Sequestosome-1 (SQSTM1), neutrophil cytosol factor 2 (NCF2), ribonuclease A family member 2 (RNASE2), and GATA Binding Protein 1 (GATA1) have been reported to mediate bacterial innate immune response [46,47,48,49,51]. SQSTM1 promotes selective autophagy to eliminate bacteria [51], while RNASE2 degrades bacterial PAMPs to allow signaling via TLR8 [52]. Common bacteria eliminated by combined SQSTM1 and RNASE2 actions include *Salmonella, E. coli, Streptococcus,* and *Mycobacterium tuberculosis* [51,52]. Decreased SQSTM1 expression has been reported to compromise the antibacterial innate immune response [48,49]. In contrast, RNASE2 is upregulated in patients with various chronic inflammatory diseases [53,54], and its expression is mediated by the transcription factor GATA1 [55]. We found that BYBG increased SQSTM1 expression at weeks 2 and 4, potentially improving antibacterial innate immunity. Both RNASE2 and GATA1 were elevated at baseline prior to BYBG, and their expression was maintained until week 4. While the change did not reach significance, both RNASE2 and GATA1 expression decreased with BYBG at week 6. Interestingly, these decreases occurred only after SQSTM1 was increased at weeks 2 and 4. Given this observed change, it is reasonable to speculate that SQSTM1 responds more rapidly to BYBG than RNASE2 or GATA1. Regardless of the response pattern, the combination of the responses of SQSTM1, RNASE2, and GATA1 to BYBG supplementation may improve the innate immune capacity to respond to future bacterial infections, which is a hallmark of innate immune training.

We found that the expression of both MX dynamin-like GTPase 2 (MX2) and interferon-induced proteins with tetratricopeptide repeats (IFIT1) was increased with BYBG, likely reflecting an improved antiviral immunity [47,56]. Specifically, MX2 is an interferon-stimulating gene that activates antiviral aspects of the innate immune response against HIV-1 [47]. Given the principles of trained innate immunity, the observed increase in MX2 expression with BYBG at weeks 2 and 4 represents an increased ability of the innate immune system to respond to a variety of viral challenges. IFIT1 allows the innate immune system to identify viral RNA and inhibit subsequent replication of that RNA [56]; thus, our observed increase with BYBG at weeks 2 and 4 may represent an improved ability to suppress viral RNA replication. Overall, the observed increase in MX2 and IFIT1 expression with BYBG may improve the trained innate immune response to future viral infection.

In addition to affecting bacterial and viral responses, BYBG altered mRNA expression associated with self-antigen responsiveness, adhesion, transmigration, phagocytosis, and/or oxidative burst [57,58,59]. Signal regulatory protein A (SIRPA) encodes the phagocyte receptor that recognizes CD47 (which was not measured in the present study) on other leukocytes [59]; this receptor is referred to as the “don’t eat me” receptor because it allows the innate immune system to differentiate between live and dying/dead cells [58,59]. Given the function of SIRPA, it is reasonable to speculate that the observed increase in expression with BYBG would limit collateral damage during the innate immune response to DAMP and PAMP.

Selectin P ligand (SELPLG) mediates leukocyte adhesion and, while it has the highest affinity for platelet (P) selectin, can also bind endothelial (E) and leukocyte (L) selectins [60]. The increase in SELPLG expression we observed with BYBG (week 2) may represent a beneficial adaptation because it allows for increased adhesion and subsequent transmigration to the peripheral tissue compartment. Aging has been shown to reduce the expression of cadherin-1 (CDH1) and thus increase susceptibility to age-associated airway epithelial barrier dysfunction [61]. Our findings demonstrate that BYBG was associated with increased CDH1 expression, which may guard against age-associated decreases in airway epithelial barrier function.

CD32 plays a key role in neutrophil phagocytosis of *S. aureus*. Therefore, reduced CD32 expression limits the normal innate immune response [57]. Increased CD32 expression with BYBG may translate to improved neutrophil phagocytosis of bacteria. In addition to bacterial phagocytosis, it is important that neutrophils can generate sufficient oxidative bursts to destroy ingested bacteria. Neutrophil cytosol factor 2 (NCF2) is a critical subunit of NADPH oxidase that is responsible for generating superoxide for the respiratory burst response of granulocytes [46]. We report that BYBG was associated with increased NCF2 expression at weeks 2, 4, and 6.

We previously reported that various strenuous running efforts acutely increased the expression of C-X-C chemokine receptor (CXCR1) expression [30], which makes sense given its known role in the mediation of IL-8-induced inflammation [62]. We found that BYBG increased CXCR1 expression (week 2), improving innate immune response to various molecular patterns (i.e., DAMP or PAMP) in subsequent weeks. We also previously reported that BYBG increases postexercise expression of TNFSF10 [28], and the present findings further demonstrate that BYBG also increases TNFSF10 expression after 4 weeks of supplementation. Sedentary adults tend to have a lower circulation TNSF10 concentration [63]; thus, the observed BYBG increase in TNFSF10 expression may counter lifestyle-associated increase.

Natural killer cell group 7 sequence (NKG7) is a common target for inflammation reduction treatments in patients with chronic diseases [64]. A reduction in NKG7 at week 6 may be a beneficial response that helps to guard against the accumulation of systemic inflammation. Integrin beta 7 (ITGB7) mediates leukocyte transmigration to gut-associated lymphoid tissue [65] during chronic disease, but we are uncertain what, if anything, our observed decreased expression with BYBG in healthy individuals may mean. Collectively, the observed changes in CXCR1, TNFSF10, NKG7, and ITGB7 expression with BYBG in healthy individuals may have implications for exercise response and the management of systemic inflammation.

### 4.4. PAMP, DAMP, and Inflammatory Response

The ability to initiate a danger response and inflammatory signal to exogenous (PAMP) and endogenous (DAMP) molecular patterns is important to the response to infection and sterile injury, respectively [66,67,68,69,70,71]. The innate immune system increases its adaptation via repeated exposure to PAMP/DAMP to maintain a trained innate immune response. The BYBG used in the present study represents one means by which to train the innate immune system and cause specific changes in mRNAs associated with PAMP/DAMP signaling and/or the inflammatory response [32,33,34,35,36].

Myeloid differentiation primary response 88 (MYD88) regulates innate immune cell detection and signaling following exposure to PAMP and DAMP [66,67,68]. Our laboratory has previously reported that BYBG supplementation increased MYD88 expression after an exercise session [28], and the present study expands that finding by revealing increased expression of MYD88 after 4 weeks of BYBG supplementation in the absence of an exercise stressor.

Interferon regulatory factor 7 (IRF7) is a downstream component of the DAMP/PAMP signaling pathway [69], and junctional adhesion molecule A (F11R) is a PAMP receptor for LPS, peptidoglycan, mannose, beta glucan, and other microorganisms and bacteria [69]. In addition to IRF7 and F11R, tissue transglutaminase (TG2) also mediates the inflammatory response to PAMP/DAMP signals via the STING pathway [72]. The increases in IRF7, F11R, and TG2 with BYBG represent a beneficial increase in capacity to respond to DAMP/PAMP.

A reduction in CD87 and CD97 expression on granulocytes/monocytes is associated with increased systemic inflammation [73,74]. BYBG increased CD87 and CD97 expression (weeks 2 and 4), likely reflecting a reduced systemic inflammatory capacity. V-domain Ig suppressor of T-cell activation (VISTA), GTP-binding protein (RHOG), and PYD and CARD domain containing (PYCARD) are all associated with various aspects of T-cell activation, mobilization, and the inflammatory response [75,76,77]. In the present study, VISTA, RHOG, and PYCARD expression increased with BYBG at week 2, which may reflect an optimized T-cell inflammatory response to future PAMP/DAMP challenge.

ADAM metallopeptidase domain 8 (ADAM8) is a metalloprotease important for the removal of damaged skeletal muscle following sterile inflammation [71]. An increase with BYBG at week 2 represents an increased capacity to respond to a subsequent exercise stimulus. Previous research has reported that strenuous exercise and subsequent sterile injury increase the release of CCL4 and CCL5 and are positively correlated with inflammation [78]. Thus, our observed decrease in CCL4 and CCL5 expression in response to BYBG at week 6 may translate to improved optimization of the inflammatory response to future exercise challenges. Indoleamine 2,3-dioxygenase 1 (IDO1) is an immune checkpoint protein that, when induced by IL-1beta, perpetuates an inflammatory signal [79]. A BYBG-induced decrease in IDO1 expression (week 6) represented a trained innate immune adaptation. Tumor necrosis factor (TNF-α) promotes inflammation (when bound to TNF receptor 1; TNFR1) or tissue recovery (when bound to TNF receptor 2; TNFR2) [80]. Our observed increase in both TNFR1 and TNFR2 expression in response to BYBG at week 2 may represent a trained immunity effect increasing the capacity to respond to sterile injury and the subsequent inflammatory response. We speculate that the changes observed with BYBG in terms of PAMP/DAMP and the inflammatory response may be novel indices of trained innate immunity. Additional research will be needed to mechanistically determine how BYBG supplementation results in the observed changes.

### 4.5. Study Limitations

The dosing strategy, on the surface, may seem like a limitation; however, based on previously published studies, it appears that BYBG doses between 50, 125, and 250 mg/day yield very similar changes [15,16,17,18,19,20,28]. Thus, we speculate that the observed changes in mRNA expression are reflective of duration of supplementation rather than dose of supplementation. Our study objective was to discover mRNA impacted by BYBG during 6 weeks of supplementation. Now that we have completed that objective, future studies could seek to conduct additional comparisons of BYBG dose or kinetic effects if that were warranted. Given previously similar responses between 50, 125, and 250 mg/day, we speculate that additional investigations of dose or kinetic effects is unlikely to yield additional clinically relevant information. The goal of BYBG supplementation is to train the innate immune system in a clinically relevant manner, which is what the key findings of this study reflect using mRNA expression biomarkers.

Any study with many mRNA expression targets may be prone to detect significance that is chance and not a real effect. Our approach to guarding against this source of false discovery is described above in Section 2.6. Another source of variability with respect to mRNA expression relates to total and differential leukocyte concentrations, which were not significantly different between sampling points in the present study. This lack of change in leukocyte concentration is relevant because it means the observed findings may not simply be due to a redistribution of leukocyte subpopulations. Additional work will be needed in the future to further validate and refine a resting BYBG mRNA signature. This may result in the invalidation of targets identified in the present study or addition of new targets that were missed by false negatives. It is our hope that the present study will serve as a foundation for additional research by our laboratory and others to fully validate the effect of BYBG on resting mRNA response in people.

## 5. Conclusions

Using previously described procedures [27,29], we were able to identify that oral BYBG supplementation uniquely altered the expression of 40 mRNAs associated with the innate immune system. Furthermore, we were able to group the responding mRNAs and identify eight specific innate immune-related pathways. Together, the response of the 40 mRNAs and the eight enriched pathways may define a BYBG "mRNA signature representing a coordinated response that prepares the body for subsequent systemic challenges. The identification of a BYBG-induced response pattern corroborates with previously published studies from our laboratory and others [14,15,16,17,18,20]. In previous studies, it was necessary to expose the human subject to an endogenous stressor (i.e., exercise) to confirm that innate immune training had occurred because traditional immune response biomarkers are minimally changed at homeostasis. The novel findings of these study are significant because, to our knowledge, we are the first to report what appears to be innate immune training in the absence of a physical stressor. The observed responses could only be detected because of the precision and low measurement error of the NanoString mRNA detection method; this would not be possible using protein biomarkers (although we recognize that mRNA and protein biomarkers yield different information concerning a given intervention) [21,22,23,24].

Our findings complement and extend previously published research reporting that BYBG improves the infection response via similar innate immune system pathways, as observed in the present study [14,15,16,17,18,19]. Another unique aspect of the present findings is that the BYBG signature was present at rest in the absence of PAMP/DAMP stimuli. Interestingly, the largest number of changes occurred after 2 weeks of BYBG, with fewer changes occurring at weeks 4 and 6. Future research may seek to evaluate intermittent/flexible dosing and the kinetics of response to better understand how BYBG trains the innate immune system for improved future response. The approach used in this study may also serve as the basis for future research conducted in free living individuals supplementing with BYBG.

## 6. Practical Applications and Generalizability of Results

The key findings of the present study were generated based on changes in the expression of systemic mRNA biomarkers in humans consuming baker’s yeast beta-glucan (BYBG) at rest. As a reader, it might not be immediately obvious how one might generalize or apply the observed effects to human health. We would urge you to not take these findings alone but rather consider them in conjunction with our previously published BYBG studies [14,15,16,17,18,19]. Specially, our previous studies have demonstrated that BYBG supplementation is effective at boosting post-exercise immune response via changes in circulating cytokine proteins and the activation status/function of leukocytes. In our previous models, exercise (i.e., sterile inflammation) was used as a memetic for an exogenous infection. This type of perturbation model was previously necessary because traditional immune response biomarkers are not sensitive enough to detect small changes in innate immune training under homeostasis. Our laboratory has been using multiplex mRNA biomarkers for many years now and found them to have unique precision and sensitivity advantages over traditional immune biomarkers. It is not often that a hypothesis is proven true, but in the case of the present study, we found that BYBG uniquely changed the expression of 40 mRNAs attributed to innate immune response. We anticipate that this is the first of many studies from our laboratory using precise mRNA biomarkers to elucidate the impact of dietary supplements like BYBG. This approach is routinely used in drug discovery, and it seems fitting to be used in further elucidation of the effects of dietary supplements on human health.

## Figures and Tables

**Table 1:** Pathway enrichment for BYBG associated mRNA changes.

Pathway	Baseline	Week 2	Week 4	Week 6
Interferon Signaling	1.006 *	1.283 *	1.387 *	1.046 *
Cell Migration and Adhesion	1.062 *	1.357 *	1.248 *	1.195 *
Extracellular Matrix Remodeling	0.982	1.491 *	1.271 *	1.047 *
Cytokine Signaling	0.912	1.206 *	1.221 *	0.913
TLR Signaling	1.125 *	1.226 *	1.283 *	1.199 *
Antigen Presentation	1.061 *	1.174 *	1.272 *	1.074 *
Metabolism	0.966	1.239 *	1.200 *	1.036 *
Complement Activation	1.303 *	1.676 *	1.598 *	1.472 *

Values represent Global Significance Scores (GSS). * Indicates significant pathway enrichment at GSS > 1.000.

## Data Availability

The data presented in this study are available on request from the corresponding author (privacy reason).
